# Epidemiological and Clinicopathological Features of *Anaplasma phagocytophilum* Infection in Dogs: A Systematic Review

**DOI:** 10.3389/fvets.2021.686644

**Published:** 2021-06-23

**Authors:** Sarah El Hamiani Khatat, Sylvie Daminet, Luc Duchateau, Latifa Elhachimi, Malika Kachani, Hamid Sahibi

**Affiliations:** ^1^Department of Medicine, Surgery and Reproduction, Hassan II Institute of Agronomy and Veterinary Medicine, Rabat, Morocco; ^2^Department of Companion Animals, Faculty of Veterinary Medicine, Ghent University, Ghent, Belgium; ^3^Department of Comparative Physiology and Biometrics, Faculty of Veterinary Medicine, Ghent University, Ghent, Belgium; ^4^Department of Pathology and Veterinary Public Health, Unit of Parasitology, Hassan II Institute of Agronomy and Veterinary Medicine, Rabat, Morocco; ^5^College of Veterinary Medicine, Western University of Health Sciences, Pomona, CA, United States

**Keywords:** canine granulocytic anaplasmosis, *Anaplasma phagocytophilum*, dogs, epidemiology, tick-borne disease, zoonosis

## Abstract

*Anaplasma phagocytophilum* is a worldwide emerging zoonotic tick-borne pathogen transmitted by *Ixodid* ticks and naturally maintained in complex and incompletely assessed enzootic cycles. Several studies have demonstrated an extensive genetic variability with variable host tropisms and pathogenicity. However, the relationship between genetic diversity and modified pathogenicity is not yet understood. Because of their proximity to humans, dogs are potential sentinels for the transmission of vector-borne pathogens. Furthermore, the strong molecular similarity between human and canine isolates of *A. phagocytophilum* in Europe and the USA and the positive association in the distribution of human and canine cases in the USA emphasizes the epidemiological role of dogs. *Anaplasma phagocytophilum* infects and survives within neutrophils by disregulating neutrophil functions and evading specific immune responses. Moreover, the complex interaction between the bacterium and the infected host immune system contribute to induce inflammatory injuries. Canine granulocytic anaplasmosis is an acute febrile illness characterized by lethargy, inappetence, weight loss and musculoskeletal pain. Hematological and biochemistry profile modifications associated with this disease are unspecific and include thrombocytopenia, anemia, morulae within neutrophils and increased liver enzymes activity. Coinfections with other tick-borne pathogens (TBPs) may occur, especially with *Borrelia burgdorferi*, complicating the clinical presentation, diagnosis and response to treatment. Although clinical studies have been published in dogs, it remains unclear if several clinical signs and clinicopathological abnormalities can be related to this infection.

## Introduction

Canine granulocytic anaplasmosis (CGA) is an emerging zoonotic tick-borne disease that is distributed worldwide. The causative agent, *Anaplasma phagocytophilum*, is an obligate intracellular gram-negative alpha-proteobacterium that develops within granulocytic cells. It is usually transmitted by ticks belonging to the genus *Ixodes* and it causes disease in several mammalian species (1, 2). In the USA, both canine and human exposures have progressively increased from 2008 to 2010 with the number of reported human cases increasing by 53% during this period (3, 4). Data from the USA Center for Disease Control and Prevention (4) and Morbidity and Mortality Weekly Report (MMWR) reported 36,342 human cases between 2010 and 2018 and almost a 12-fold increase during this same period (4). Currently, human granulocytic anaplasmosis (HGA), is considered amongst the three most important vector-borne disease (VBD) in the USA with Lyme borreliosis and Zika virus (5, 6) and is increasingly being diagnosed in several European and Asian countries (7, 8).

The focus on canine VBDs has increased the past decade as they represent an important threat to both canine and human health (9). Because of their proximity to humans, dogs may serve as reservoirs of vector-borne pathogens, a source of infection for vectors, mechanical transporters of infected vectors, and as sentinel indicators of regional infection risk (2, 3, 10–15). Furthermore, the strong molecular similarity between human and canine isolates of *A. phagocytophilum* in Europe and the USA (16–21) and the positive association in the distributions of human and canine cases in the USA emphasizes the use for dogs as sentinels in epidemiological studies (3, 4, 9, 15, 22, 23).

The lack of specific clinicopathological signs, the frequent rapid evolution and positive prognosis even without treatment, the prompt response to a commonly used antibiotic and the possibility of coinfections (24–28) all make the diagnosis of CGA challenging for veterinarians. Description of signs and laboratory abnormalities associated with *A. phagocytophilum* infection in dogs is mostly available from Europe and North America. Although some studies have described the most common manifestations of CGA (13, 24–35), it remains unclear if some clinical signs and clinicopathological abnormalities are related to this infection. In this paper, we provide an overview of the current knowledge on the worldwide epidemiological features of *A. phagocytophilum* focusing on dogs, and describe the clinicopathological aspects of CGA with an emphasis on missing data.

## Description of *Anaplasma phagocytophilum*

### Classification

*Anaplasma phagocytophilum* is a bacterium belonging to the family of Anaplasmataceae in the order of Rickettsiales (36). The phylogenetic molecular analysis based on the 16S rRNA and the groEL genes sequencing in addition to morphologic and phenotypic characteristics have led to the reorganization of the family of Anaplasmataceae and the reclassification of some agents. Consequently, the name *A. phagocytophilum* was given in 2001 to three previously distinct agents, i.e., the agent that causes equine granulocytic anaplasmosis (*Ehrlichia equi*), the agent that causes tick-borne fever or pasture fever in sheep and cattle (*Ehrlichia phagocytophila*) and the agent that causes HGA [formerly human granulocytic ehrlichiosis (HGE)] (1). The renaming of these three agents as *A. phagocytophilum* has been controversial because of differences in their host tropism and cell target from other *Anaplasma* species, such as *Anaplasma marginale* (37). Additionally, although these three agents share genetic, antigenic and biological characteristics (1), they are considered phenotypic variants due to differences in their distribution, prevalence, virulence and target host species (38, 39).

### Morphology and Genome

*Anaplasma phagocytophilum* typically exhibits coccoid to ellipsoid shapes measuring ~0.2–2.0 μm in diameter. The bacteria infect myeloid cells primarily neutrophils (and occasionally eosinophils), forming intracytoplasmic inclusions derived from the host cell membrane measuring 1.5–2.5 μm, called “morula” (from Latin “morum”: mulberry) (1, 40).

The *A. phagocytophilum* genome is composed of a single circular double-stranded chromosome. The complete genomic sequence is estimated at 1.47 megabases (Mb) and was published on GenBank in 2006 (NC007797) (19, 36). Despite its apparently simple genome, *A. phagocytophilum* exhibits an extensive genomic diversity (19, 41, 42). More than 500 partial *A. phagocytophilum* pseudogene sequences derived from human, ticks and animals from several US, European and Asian regions are available in GenBank (19). Moreover, twenty complete *A. phagocytophilum* genomes have been sequenced including 16 American and four European strains. However, genomes from only a few different strains per host species are available (aside from humans), underscoring the lack of information on strain diversity within different host species (19, 36).

### Genetic Variability

Genetic variability between strains may explain the ecological complexity, the host tropism diversity, the differences in incidence and clinical presentation, severity and evolution of the disease documented in different countries (42–47). Many studies demonstrated different virulence and hosts tropism of specific *A. phagocytophilum* strains (17, 41–50). However, the host specificity of strains seems to be restricted and multiple infections with different strains are often observed. Farm and large wild animals, small mammals and ticks were especially prone to carrying multiple genetic variants. In humans and domestic animals double infections are not so frequent (51). The 16S rRNA gene nucleotide sequences analysis discriminated 15 worldwide variants differing in a variable fragment located near the 5' end of the gene. Among them, two are pathogenic for human and abundant all over the world (52). In the USA, several variants have been identified based on the sequencing of the 16S rRNA and the only pathogenic variant to humans (AP-ha) is also able to induce the disease in dogs, horses and mice but not in cattle. In Europe, other variants have been identified in humans and the AP-ha variant was also detected in wild ruminant species (41–43, 48, 49). Strains infecting domestic ruminants in Europe and white-tailed deer in the USA seem to genetically differ from those infecting humans, horses and dogs (44, 50). In Washington State, five different 16S rRNA variants (named WA1–5) that differed at four nucleotide positions were identified from dogs displaying clinical signs consistent with CGA. All WA variants were distinct from those identified in sheep in Norway and llama-associated ticks but one was identical to equine and human variants (24). In another European study, seven different 16S rRNA variants were identified from dogs, with the two most common variants showing statistically significant differences in the frequency of clinical signs and hematological abnormalities, which suggests possible differences in strain pathogenicity (45). Finally, a recent study showed that dogs can be naturally infected concurrently with *A. phagocytophilum* variant 1, variant 4, and HGE agent (53). The pathogenic role of the classic sheep variant, *A. phagocytophilum* variant 1, in the canine species is uncertain. Previous studies showed that the “HGA agent” appears to be more pathogenic for dogs than other variants (45).

The 16S rRNA gene was considered too conserved for use in the phylogenetic analysis of different strains of *A. phagocytophilum*. It has a poor resolution and failed to discriminate between ecotypes circulating in wild ruminants compared to other animals. Furthermore, the 16S rRNA sequence analysis could not categorize human-infective isolates in order to detect virulent strains and was unable to distinguish variants according to their geographic origin (43, 54–56). As such, other genes have been proposed to study the genetic variability of *A. phagocytophilum* including msp4, ankA, groEL operon, msp2/p44, pfam01617 superfamily, and drhm genes (19–21, 50, 56–59). Sequencing different genes revealed similarities between human and canine isolates, suggesting that dogs and humans may be infected by the same strains (16–21, 24, 45, 53, 60–62).

## Vectors

Although several transmission modes have been reported (mostly in humans) (63–66), *A. phagocytophilum* is commonly transmitted to people and domestic animals through tick bites (67). It is naturally maintained in complex and poorly understood enzootic tick-wild animal cycles (55, 59, 68) and is transmitted most frequently by ticks of the *Ixodes persulcatus* complex. These ticks are commonly found in the northern hemisphere and their occurrence depends on climatic conditions (between 10 and 30°C, and >80% relative humidity) and the availability of hosts (49, 69).

In the USA, several ixodid ticks transmit this pathogen, depending on the geographic location. The main vector in the humid forests of the upper midwestern, north central and northeastern regions is *Ixodes scapularis* whereas *Ixodes pacificus* is located in shrub forests and deserts of the western USA (70–72). The prevalence of *A. phagocytophilum* DNA among ticks varies from <1% up to 50% throughout the country (73–76). Other tick species have been reported to be infected with *A. phagocytophilum*, such as *Amblyomma americanum* and *Dermacentor* spp., and *Ixodes spinipalpis* and *Ixodes dentatus* are recognized as competent vectors (77–81). Other *Ixodes* species including *Ixodes angustus, Ixodes ochotonae*, and *Ixodes woodi* are suggested to act as vectors for the bacterium (82, 83). In central and southern America, very few studies are published on the prevalence of *A. phagocytophilum* among ticks. However, among the three available studies, none have detected the DNA of this bacterium in *Ixodes* spp. ticks. In contrast, its DNA has been amplified from *Rhipicephalus sanguineus, Amblyomma cajennense, Amblyomma dissimile, Amblyomma maculatum, Dermacentor variabilis* (84–86). *Amblyomma* spp. and *D. variabilis* were positively correlated with *A. phagocytophilum* infection in Brazil and Mexico (84, 86).

In Europe, the most common vector is *Ixodes ricinus* (69), which is widely distributed from western Europe to central Asia. This tick lives mostly in humid wooded habitats and pastures and is rarely encountered in the Mediterranean region or in mixed or deciduous forests except at high altitudes (67). The prevalence of *A. phagocytophilum* DNA among *I. ricinus* ticks in Europe varies from <1 to 76.7% (87, 88). Other *Ixodes* spp. ticks seem to be involved in epidemiological cycles that are distinct from those involving *I. ricinus* (55, 89, 90). In addition, the DNA of this bacterium has been detected in several other tick species in Europe including *Dermacentor reticulatus, Haemaphysalis concinna, Hyalomma marginatum, Ixodes ventaloi*, and *Ixodes trianguliceps* (58, 91–95). *Rhipicephalus* species were also infected by *A. phagocytophilum* and could act as competent vectors in the eastern Mediterranean area (96–99). *Ixodes persulcatus* is another competent vector of *A. phagocytophilum* in eastern Europe and Asia, with rates of DNA detection up to 16.7 and 21.6%, respectively (100, 101).

Although *I. persulcatus* is considered the primary vector in Asia, *A. phagocytophilum* DNA has been detected in several other tick species including *Ixodes nipponensis, Ixodes ovatus, Rhipicephalus turanicus, Rhipicephalus haemaphysaloides, H. marginatum, Boophilus kohlsi, Dermacentor silvarum*, and several *Haemaphysalis* species (96, 102–106). Molecular investigations indicated that *I. ovatus, Dermacentor silvarum, Hae. concinna, Haemaphysalis longicornis, Rhipicephalus microplus, R. sanguineus*, and *Dermacentor nuttalli* might be involved in the transmission of *A. phagocytophilum* in China (8, 107–109).

In North Africa, one study in Morocco and Tunisia detected *A. phagocytophilum* DNA in 1 and 3% of *I. ricinus* and *Hyalomma detritum*, respectively (110). Two separate studies detected DNA in *R. sanguineus* from free-roaming dogs in Egypt and *H. marginatum* from horses in Tunisia with prevalence rates of 13.7 and 2.3%, respectively (111, 112). These studies indicate that *A. phagocytophilum* is likely to circulate in a wide variety of ticks, but their involvement in transmitting the bacterium to host has yet to be established (112).

## Distribution and Prevalence of *Anaplasma phagocytophilum*

*Anaplasma phagocytophilum* has a worldwide distribution and endemic areas include some regions of the USA (northeastern and mid-Atlantic, Upper Midwest, and Pacific Northwest states), Europe and Asia (China, Siberian Russia, and Korea). These regions correspond to occurrence areas of *I. persulcatus* group ticks (12, 13, 24, 29, 113). Several prevalence studies in dogs have been conducted in various American, European, Asian and African countries (Table 1). However, data are lacking in large parts of Asia, Africa, South America and Australia. The geographic variation in tick exposure, the differences in inclusion criteria to select dog populations, and the use of different serologic tests [i.e., immunofluorescent antibody test (IFAT), enzyme-linked immunosorbent assay (ELISA) or Western blot] make comparison between studies difficult (234, 235). In addition, cross-reactivity with the most important other *Anaplasma* species infecting dogs, i.e., *Anaplasma platys*, is reported to occur for both IFA and ELISA (1, 9, 120, 121, 236–241). Therefore, in regions where both pathogens could be present (southern USA states, southern Europe, South America, Asia, and Africa), seropositivity may not necessarily reflect exposure to *A. phagocytophilum* and potential overestimation of the true prevalence and distribution can occur (9, 162, 189, 198, 234, 236, 238, 241). As a result, PCR-based assays are necessary to determine which of the two agents is responsible for positive serologic test results in regions where both bacteria are present (241). In areas where the *Ixodes* tick vector is less prevalent or absent, a positive *Anaplasma* spp. serologic result could be the result of *A. platys* exposure (164). Less frequent and minor serological cross-reactions were described at low titers between *A. phagocytophilm* and *Ehrlichia* species (i.e., *Ehrlichia canis, Ehrlichia chaffeensis, Ehrlichia ewingii*, and *Neorickettsis sennetsu formerly Ehrlichia sonnetsu*), especially with hyperimmune sera, when using IFA and immunoblot assay (1, 29, 39, 121, 127, 242, 243). However, it is not clear whether the cross-reactivity with *E. canis* was attributable, in part, to antibodies against *A. platys* because dogs are sometimes exposed to both *E. canis* and *A. platys* (164, 240). In contrast, no cross-reactivity has been documented between *Anaplasma* spp. and *Ehrlichia* spp. when using the point-of-care dot ELISA (234, 240).

**Table 1:** Prevalence of *Anaplasma* spp. (*A. phagocytophilum* and *A. platys*) antibodies and/or DNA detection of *A. phagocytophilum* in blood samples from dogs in several countries.

**Countries**	**Number of dogs**	**Type of dog population**	**Prevalence (%)**	**Method**	**References**
**AMERICA**					
**Canada**	86,251	Sick and healthy dogs from 238 practices	0.2	ELISA	(114)
	115,636	Not stated	0.29	ELISA	(115)
	753,468	Not stated	0.4	ELISA	(116)
7 provinces	285	Not stated	1.1	ELISA	(3)
South Ontario, Quebec	53	Suspected to have TBD	0.0	IFA	(117)
Saskatchewan	515	Sick and healthy client-owned dogs	0.6	ELISA	(118)
**USA**	3,950,852	Not stated	3.8	ELISA	(15)
	3,588,477	Sick and healthy dogs tested for VBD	4.4	ELISA	(23)
	479,640	Dogs suspected to have a VBD	4.8	ELISA	(9)
	14,496	Dogs suspected to have a VBD	1.9	ELISA	(119)
	6,268	Dogs suspected to have a VBD	1.5–3.5	ELISA	(3)
Oregon, California	2,431	Clinically healthy dogs	2.4	ELISA	(120)
North Carolina, Virginia	1,845	Dogs admitted regardless of the reason for examination to the NCSU-VTH	1.1	IFA	(121)
Maine	1,087	Dogs tested for heartworm or undergoing surgery	7.1	ELISA	(122)
California	1,385	Non-ehrlichial related illnesses or well-animal care	8.7	IFA	(10)
	184	Rural dogs with or without clinical signs	40.0 7.6	IFA PCR	(12)
Minnesota	731	Sick and healthy pet dogs	29.0	ELISA	(13)
	273		9.5	PCR	
Oklahoma	259	Dogs suspected to have a VBD	33.0	IFA	(123)
Northern Arizona	233	Pet and stray dogs	11.6 0.0	ELISA PCR	(124)
New Jersey	202	Healthy dogs	9.4	ELISA	(125)
North Carolina	118	Clinically healthy dogs	0.0	ELISA	(126)
Connecticut, New York	106	Sick client-owned dogs living	9.4	IFA, WB	(127)
Cumberland Gap Region	232	Shelter dogs	0.9	ELISA	(128)
**Brazil**					
Rio de Janeiro	398	Not stated	6.0	PCR	(84)
	253	Not stated	7.1	PCR	(129)
Southeastern	198	Dogs suspected to have TBD	0.0	PCR	(130)
Southern	196	Companion dogs	9.7	ELISA	(131)
Central-northern Parana	138	Rural and urban dogs	13.8	ELISA	(132)
**Puerto Rico**	629	Dogs from shelters and a veterinary clinic	1.0	ELISA	(133)
**Colombia**	498	Not stated (abstract only)	33.0	ELISA	(133)
	218	Working, shelter and client-owned dogs	53.2	ELISA	(134)
**Haiti**	210	Owned dogs	17.6 0.0	ELISA PCR	(135)
**West Indies**	157	Not stated	10.8	ICG	(136)
**Caribbean region**	29	Not stated	10.0	ELISA	(3)
**Mexico**	1,706	Healthy dogs and dogs with clinical signs compatible with VBD	9.9	ELISA	(137)
**Costa Rica**	408	Apparently healthy dogs	2.7	IFA	(138)
	374		0.3	PCR	
**Ecuador Galapagos Islands**	58	Without consideration of the patients' presenting complaint	12.1	ELISA	(139)
**Nicaragua**	329	Dogs presented at veterinary clinics	28.6 2.2	ELISA PCR	(140)
**Chile**	905	Urban and rural dogs	44.0	IFA	(141)
**EUROPE**					
**Germany**	5,881	Sick dogs suspected to have anaplasmosis	21.5	ELISA	(142)
	1,124	Dogs suspected to have anaplasmosis	50.1	IFA	(143)
	522	Healthy dogs and dogs suspected of CGA	43.0 5.7	IFA PCR	(144)
	111	Healthy dogs and dogs suspected of CGA	43.2 6.3	IFA PCR	(31)
	1,862	Traveling dogs to Germany	17.8	IFA	(145)
	792	Retrospective analysis of serum sample	41.9	IFA	(146)
Munich	448	Healthy and sick dogs	19.4	ELISA	(147)
	171	Healthy Bernese Mountain Dogs	50.3	IFA	(148)
	57	Healthy dogs from other breeds	24.6		
Brandenburg	1,023	Blood samples from veterinary clinics or a commercial diagnostic laboratory	1.5	PCR	(149)
**Russia**					
European part	440	Urban dogs with a history of tick bites	1.1	ELISA	(150)
Voronezh Reserve	82	Dogs owned by Voronezh Reserve staff	34.1	ELISA	
**Hungary**	1,305	Healthy pet dogs	7.9	ELISA	(151)
Southern Hungary	126	Shepherd, hunting and stray dogs	11.0	PCR	(152)
**Slovakia**	87	Dogs suspected to have babesiosis	8.0	PCR	(153)
		Dogs randomly selected	11.7	ELISA	(154)
**Bulgaria**					
Central-southern	167	Dogs presented for various clinical reasons	19.2	IFA	(155)
**Austria**	1,470		56.5	IFA	(156)
**United Kingdom**	120	Dogs suspected to have TBD	0.8	PCR	(157)
**Sweden**	611	Dogs not clinically suspected to be infected by *Ehrlichia* spp or *B. burgdorferi* sensu lato	17.7	IFA	(158)
	100	Not stated	17.0	IFA	(159)
**Finland**	340	Pet dogs with or without clinical signs of illness	5.3	ELISA	(160)
	50	Healthy hunting dogs	4.0		
**Albania**	30	Clinically healthy semi-domesticated dogs	40	IFA	(161)
Tirana	602	Client-owned dogs	24.1 1.0	IFA PCR	(162)
**Latvia**	470	Healthy dogs and dogs suspected to have borreliosis and/or anaplasmosis	11.4	ELISA	(163)
**Romania**	1,146	Guard, pet, shelter, stray and hunting dogs	5.5	ELISA	(164)
	29	Pet and stray dogs from Romania	7.4	IFA	(165)
	109	Dogs imported from Romania to Germany	2.2	PCR	
Eight counties	357	Not stated	5.3	PCR	(166)
Southeastern	257	Not stated	6.2	PCR	(167)
South Central	149	Asymptomatic shelter dogs	3.3	ELISA	(168)
**Serbia**					
Vojvodina province	84	Randomly selected dogs	15.5	IFA	(169)
Belgrade municipalities	111	Shelter, free-roaming and hunting dogs	28.8 0.0	ELISA PCR	(170)
**Poland**	3,094	Healthy dogs with a history of tick bite	12.3	ELISA	(171)
Eastern	400	Healthy dogs	8.0 2.8	ELISA PCR	(172) (173)
Northwestern	192	Dogs from endemic regions of borreliosis	1.0	PCR	(174)
	100	Healthy dogs from a shelter	4.0	PCR	
	92	Dogs suspected to have Lyme disease	14.0	PCR	(175)
	50	Dogs diagnosed with babesiosis	0.0		
	79	Apparently healthy sled dogs	1.3		
**Czech Republic**	296	Healthy dos and dogs suspected to have TBD	3.4 26.0	PCR IFA	(176)
**Italy**					
Stretto di Messina	249	Outdoor-kennel dogs Not stated	38.0	IFA	(177)
	5,881		32.8	IFA	
Central Italy	1,965	Urban and rural dogs without signs of TBD	4.7	IFA	(178)
	1,232	Not stated	8.8	IFA	(179)
	215	Hunting dogs	14.8 0.9	IFA PCR	(180)
	1,026	Owned dogs	3.3	IFA	(181)
Sicily	344	Pet, pound and hunting dogs	0.0	PCR	(182)
	87	Not stated	44.8 0.0	IFA PCR	(183)
	372	Not stated	4.8	PCR	(184)
Southern	165	Dogs with febrile illness and healthy controls	37.8 0.0	IFA PCR	(185)
Northeastern	488	Privately-owned canine blood donors and free-roaming dogs	3.3 0.0	IFA PCR	(186)
Sardinia	50	Dogs suspected of tick bite–related fever	6.0	PCR	(187)
**Portugal**	1,185	Healthy dogs and dogs suspected to have VBD	4.5	ELISA	(188)
	55	Dogs suspected to have TBD	54.5 0.0	IFA PCR	(189)
	55	Dogs suspected to have TBD	55.0	IFA	(190)
	100	Apparently healthy military dogs	16.0	IFA	(191)
**France**	919	Not stated	2.7	ELISA	(192)
**Spain**	466	Sick and healthy dogs	11.5	IFA	(11)
Nothwestern	1,100	Dogs presented to veterinary clinics	3.1	ELISA	(193)
	479		5.0	IFA	(194)
Northern	556	Healthy dogs and dogs with signs compatible with VBDs	1.26	ELISA	(195)
Central	131	Shelter dogs	19.0	ELISA	(196)
**Turkey**	757	Stray, shelter and pet dogs	0.5	PCR	(197)
Thrace region	400	Healthy shelter dogs	6.0	PCR	(198)
**Croatia**	1,080	Apparently healthy dogs	0.3	PCR	(199)
	435	Apparently healthy owned and shelter dogs	6.21	ELISA	(200)
**Greece**	200	Owned and shelter dogs	1.0 0.5	ELISA PCR	(201)
**ASIA**					
**Japan**	154	Sick and healthy dogs	0.0	PCR	(202)
	332	Dogs presented at 6 private veterinary clinics in Ibaraki Prefecture	2.1 0.3	IFA PCR	(203)
**China**	600	Companion, working and shelter dogs	0.5	ELISA	(204)
	234	Stray and pet dogs	13.2	PCR	(205)
	219	Dogs from rural areas	10.0 10.9	IFA PCR	(107)
	26	Dogs from rural areas	7.7 50	ELISA IFA	(206)
	562	Dogs presented for reasons unrelated to suspicion of VBD	2.7	ELISA	(207)
	637	Apparently healthy indoor and breeding dogs	1.4	ELISA	(208)
	201	Apparently healthy stray dogs	11.9	PCR	(209)
**Korea**	1,058	Shelter dogs	0.1	PCR	(210)
	532	Outdoor dogs	15.6 2.3	ELISA PCR	(211)
	418	Shelter dogs	1.2	ELISA	(212)
	229	Urban shelter dogs and rural hunting dogs	18.8	ELISA	(213)
	245	Blood samples from military dogs	4.4 0.0	IFA PCR	(214)
**Malaysia**	48	Stray dogs	9.3 4.3	ELISA PCR	(215)
**India**	191	Pets, stray and working dogs	4.7	ELISA	(216)
	230	stray dogs in Tamil Nadu	0.4	PCR	(217)
**Israel**	195	Healthy pet dogs, stray and shelter dogs	9.0	IFA	(218)
**Jordan**	38	Stray dogs	39.5	PCR	(219)
	161	Stray, police, or breeding with tick infestation	9.9	ELISA	(220)
**Taiwan**	175	Asymptomatic dogs	21.1 0.0	ELISA PCR	(221)
**Iran**	103	Apparently healthy rural dogs	57.3	PCR	(222)
	150	Owned and stray dogs from Tehran	2.0	PCR	(223)
**AFRICA**					
**Tunisia**	286	Healthy and sick pet, kenneled dogs	25.2 0.9	IFA PCR	(224)
**Algeria**					
Algiers	150	Owned dogs admitted for surgery or vaccination	47.7	IFA	(225)
	63	Stray dogs from a shelter	0.0	PCR	
**Morocco**	217	Owned urban, rural and military healthy dogs or displaying signs of VBD	16.6	ELISA	(226)
Northwestern	425	Owned urban, rural and military healthy dogs or displaying signs of TBD	21.9 0.0	ELISA PCR	(227)
**Nigeria**	245	Healthy and sick dogs	0.8	PCR	(228)
**South Africa**	141	Apparently healthy owned and free roaming dogs	2.1	PCR	(229)
	56	Apparently healthy domestic dogs	0.3	PCR	(230)
**Ghana**	17	Client-owned dogs presented for a variety of complaints or for vaccination	11.8 0.0	ELISA PCR	(231)
**Cape Verde**					
Priai	57		1.8	PCR	(232)
Mayo Island	153	Apparently healthy dogs	0.0	PCR	(233)

*TBD, tick-borne disease; VBD, vector-borne disease; IFA, immunofluorescence assay; ELISA, enzyme-linked immunosorbent assay; PCR, polymerase chain reaction, WB, western blot; ICG, immunochromatography*.

The first CGA cases in the USA were detected in California; then, the exposure of dogs to this organism has been recorded in more than 39 USA states and highest rates were noted in the upper Midwestern, northeastern and western states. Serologic surveys revealed prevalence values of *Anaplasma* spp. antibodies ranging from 0.0 to 40.0% (3, 9, 10, 12, 13, 15, 23, 119, 120, 122–126, 236, 242, 244, 245). Five countrywide serologic studies showed an overall prevalence of *Anaplasma* spp. of 1.9 to 4.8% with the highest rates recorded in northeastern regions (3, 9, 15, 23, 119). The study that found a prevalence rate of 1.9%, used species-specific peptides to detect canine antibodies to *A. phagocytophilum* (3). In addition, cases confirmed by PCR were diagnosed in several USA states (12, 13, 24, 26, 27, 29, 124, 245–247). In the USA, over 100,000 and 220,000 dogs were seropositive to *Anaplasma* spp. in 2015 and 2019, respectively (248, 249). Two recent studies analyzing regional trends of *Anaplasma* spp. exposure in dogs showed that seroprevalence increased broadly in the northeastern, upper midwestern states, northern California, mid-atlantic coast and southern Oregon (249, 250). In Canada, six serologic surveys on *Anaplasma* spp. are available (Table 1) (3, 114–118), and six cases of CGA from Vancouver Island (251), Saskatoon (252) and Montreal (253) were confirmed by DNA detection. In Latin America and the Caribbean, the seroprevalence of *Anaplasma* spp. ranges from 1.0 to 53.2% (Table 1) (133, 134, 254). In addition, two studies and a case report have detected the DNA of *A. phagocytophilum* in Brazil (Table 1) (129, 255).

In Europe, *Anaplasma* spp. seroprevalence has been reported in almost all countries with rates ranging from 1.1 to 56.5% (143, 148, 150, 183, 190). The detection of *A. phagocytophilum* DNA has also been reported mostly from central and northern countries (Table 1) with prevalence rates up to 14.2% (174). Additionally, several cases of CGA have been described (25, 28, 30–32, 34, 256–262).

In Asia, *Anaplasma* spp. seroprevalence is available from China, Korea, Malaysia, Taiwan and Israel and range from 1.2 to 24.7% (Table 1) (212, 214). *Anaplasma phagocytophilum* DNA has also been detected in dogs with prevalence rates up to 39.5 and 57.3% in Jordan and Iran, respectively (219, 222).

In Africa, only a few prevalence studies have been published on *Anaplasma* spp. in dogs (Table 1). Seroprevalence rates recorded in African countries range from 11.8 to 47.7% (Table 1) (225, 231). Similarly, very limited studies have investigated *A. phagocytophilum* infection in dogs in this continent. The DNA of this bacterium has been detected in Tunisia, Nigeria, Cape Verde and South Africa (Table 1) (224, 228, 229, 232) but not in Algeria and Morocco (225, 227). In addition, an *Anaplasma* species closely related to *A. phagocytophilum* was detected in blood samples from South African dogs based on 16S rRNA gene sequencing (263) whereas all dogs from Algeria, Ghana and Maio Island tested negative by PCR (Table 1) (225, 231, 233).

## Epidemiological Role of Dogs

Several wild and domestic animals are receptive to *A. phagocytophilum* infection. However, the disease has been reported only in a few species including domestic ruminants, horses, cats, dogs and humans (22, 24, 63, 264–269). Although dogs are susceptible to *A. phagocytophilum* infection, they are mostly recognized as incidental hosts and their role as potential reservoirs is still controversial (24, 270). As *A. phagocytophilum* is an obligate intracellular bacterium, its reservoirs should be animal hosts permitting its survival, particularly outside the activity period of its vectors (271). To be considered as a host reservoir, a host must be fed on by an infected vector tick at least occasionally, take up a critical number of the infectious agent during the bite by an infected tick, allow the pathogen to multiply and survive for a period in at least some parts of the body, and allow the pathogen to find its way into other feeding ticks (272, 273). Therefore, the detection of pathogens or their DNA in animal hosts is not enough to consider them as reservoir hosts (274).

Dogs are considered unlikely reservoir hosts due to the probable short duration of bacteremia (<28 days) and uncertainty regarding their ability to host enough nymphal tick stages to contribute to the spread of the bacterium (2, 67). In Austria, no significant difference in the seroprevalence of *A. phagocytophilum* among owners of seropositive pets and owners without pets was observed, suggesting that pets are not a source of infection for humans (275). However, wild and domestic carnivores are considered the primary source of tick-borne zoonotic agents to humans (276) and contact with pet cats and dogs has been proposed as a risk factor for tick exposure and tick-borne disease among humans (277, 278). Moreover, according to some authors, almost all studies investigating the role of dogs in the transmission of tick-borne diseases (TBDs) focused on companion dogs. These animals are usually treated for ectoparasites, have limited free access to the outdoors and host reservoir habitats, and are less exposed to ticks compared with hunting, stray or shelter dogs. Therefore, these studies may not accurately reflect the public health risk associated with dogs in endemic areas (152). Others suggested that domestic animals including dogs could be considered as reservoir hosts of *A. phagocytophilum* in Europe especially in urban areas (18, 270, 279–282). In a study from Hungary, the prevalence of *A. phagocytophilum* DNA in stray dogs was higher than in several studies from other European countries (152). In addition, two studies reported high prevalence rates of *A. phagocytophilum* DNA in dogs suspected to have Lyme disease and rural dogs from Poland and China, respectively (107, 174). *Anaplasma phagocytophilum* was also the most frequently detected bacterium by PCR in stray dogs that lived in close contact with domestic animals and humans in rural and peri-urban areas of the Mediterranean zone of Jordan (219). In addition, high prevalence rates of *A. phagocytophilum* DNA was found in *I. ricinus* collected from dogs in Belgium and Poland, and *R. sanguineus* (adult and nymphs) from free-roaming dogs in Egypt (111, 280, 283). Moreover, *A. phagocytophilum* DNA was detected in experimentally infected dogs during 60 days without immunosuppressive drug, and the canine immune response seems to have evolved to only partially control infection, suggesting a longer bacteremia possibly allowing timely transmission to the vector (18, 284). Based on these results, dogs could act as potential reservoir for the bacterium at least in some regions, but further studies are needed.

The geographical distribution of canine infection seems to parallel the distribution of HGA in the USA with a positive association of human and canine cases in many states (3, 23). Indeed, several studies found the highest prevalence rates of *A. phagocytophilum* antibodies in dogs from the upper midwest, northeast, and mid-atlantic, which correlate with areas where the highest incidence of human anaplasmosis were reported (3, 4, 9, 15, 22, 23). In addition, the estimated regression coefficient for the endemic risk factor in the contiguous USA model was positive and significant. This implies a higher prevalence among dogs living in areas where HGA is endemic (15). Furthermore, a study has evaluated regional and local temporal trends of canine *Anaplasma* spp. (*A. phagocytophilum* and *A. platys*) exposure using a Bayesian spatio-temporal binomial regression model for analyzing serologic test results. In this study, similarity was found between temporal trends in canine *Anaplasma* spp. seroprevalence and the reported incidence rate of HGA (249). Finally, human and canine strains of *A. phagocytophilum* were similar according to several gene sequencing studies, and human isolates have been reported to induce clinical disease in dogs in both Europe and the USA (16–21). Therefore, in addition to the possible role of dogs as potential reservoir hosts, the prevalence data of *A. phagocytophilum* infection in dogs provides important information on the incidence, risk factors, exposure sources, and real-time risk of exposure for human infection (3). More generally, several studies have documented the utility of using dogs as sentinels for human vector-borne diseases (VBDs) (14, 17, 18).

## Pathogenesis of *Anaplasma phagocytophilum* Infection

*Anaplasma phagocytophilum* is transmitted by ticks to their hosts within 24–48 h of feeding time (285–288) but establishment of infections in dogs is apparently dependent on a minimum inoculation dose (288). Bacteremia, however, develops 4–7 days after the tick bite during natural infection or 3–4 days after experimental blood inoculation, suggesting that the bacterium remains at undetectable levels in the blood or replicates in other cells in the early stages of infection (69). Cell surface analysis suggested that the endothelial cells of the microvasculature provide an excellent site for *A. phagocytophilum* dissemination to peripheral blood granulocytes. Endothelial cells may play a crucial role in the development of persistent infections and are stimulated to express surface molecules and cytokines in a dose-dependent manner that may lead to inflammatory responses at the site of infection (289). After inoculation, *A. phagocytophilum* exhibits a biphasic developmental cycle in which the infectious small dense-cored cells bind to host cellular targets and enter the cytoplasm of neutrophils by endocytosis. After, the non-infectious reticulate cells multiply by binary fission within phagosomes until forming morulae. After 28–32 h, replication ceases and reticulate cells re-transition to dense-cored cells that are released after cell lysis to initiate the next wave of infection and possibly spread to multiple organs (40, 290, 291).

*Anaplasma phagocytophilum* has several strategies to dysregulate the bactericidal functions of neutrophils and ensure its survival and replication. This bacterium regulates host defense and antimicrobial mechanisms by a direct interaction with specific gene regulatory regions in the nucleus of the neutrophil, decreasing endothelial adherence, mobility, transmigration, phagocytic activity, and degranulation. It can also alter the respiratory and oxidative burst mechanism of neutrophils, delay apoptosis and increase the inflammatory recruitment of new neutrophils (289, 292–297). In addition, the antigenic variation of the immunodominant surface proteins msp2/p44 enables the bacterium to evade the specific immune response and to subvert the adaptive immune response (297). Neutrophils circulate for 10–12 h before they enter tissues and undergo apoptosis, which may lead to the destruction of the pathogens. Therefore, the decreased endothelial adherence and delayed apoptosis both enhance the bacterial survival and the replication to form morulae in a normally short-lived, terminally differentiated granulocytic cell. Furthermore, the impaired neutrophil function can result in an immune deficiency, predisposing patients to opportunistic infections (293–295). *Anaplasma phagocytophilum* was suggested to possibly manipulate the host endoplasmic reticulum stress signals to facilitate intracellular proliferation and infection of surrounding cells before or after host cell apoptosis (298).

The immune response induced by *A. phagocytophilum* is thought to play an important role in the initial control of the disease but may also induce inflammatory injuries associated with granulocytic anaplasmosis. Indeed, the absence of *A. phagocytophilum* control induces a clear rise in inflammatory lesions, which is considered the major pathogenic effect in humans and murine models (299–301). In dogs, the hematological modifications associated with *A. phagocytophilum* infection are similar to those induced by other members of *Ehrlichia* or *Anaplasma* genera, although they infect different blood cells, suggesting that the major mechanism of cytological injuries is related to an immunological response or to substances secreted from the bacteria (302, 303). *Anaplasma phagocytophilum* induces an upregulation of chemokine and pro-inflammatory cytokine [IL-8, macrophage inflammatory protein (MIP)-1a, MIP-1b, monocyte chemoattractant protein (MCP)-1] expression *in vitro*, which attracts leukocytes and inhibits hematopoiesis leading to myelosuppression (304, 305). In mice, several leukocyte populations expand during infection including NK and NKT cells followed later by CD4 and CD8 T lymphocytes and the immune response proceeds mostly through production of interferon gamma (IFN-γ), commonly produced by T lymphocytes (301–303, 306). In humans, the manifestation of severe disease is associated with hypercytokinemia and macrophage activation or hemophagocytic syndromes (MAS/HPS). The underlying pathogenesis of MAS/HPS is poorly understood; however, it is frequently associated with a defective function or depletion of cytotoxic cells and is driven mostly by the persistent stimulation of cytokine production, especially the macrophage-activating IFN-γ (307–309). The clear role of IFN-γ in the pathogenesis of the disease is demonstrated by the observation that the lack of this molecule in *A. phagocytophilum*-infected mice resolves inflammatory tissue injury (300).

Several studies have confirmed the role of the IFN-γ in mediating both the pathology and early control of bacteria, although it is not essential for bacterial clearance. Other protective mechanisms might be involved in the control of *A. phagocytophilum* infection, as some infected mice lacking IFN-γ are able to survive (292, 300–303, 306). Data suggest that the humoral immunity may also play an important role in the clearance of ehrlichial infections, as passive immunization has a moderately protective effect. Moreover, severely immunocompromised mice that lack both B and T cells remained persistently infected, as opposed to mice lacking only T cells, which were able to control the infection (301, 310). Experimentally infected dogs develop serologic responses (immunoglobulin G) 7 days after inoculation. However, positive *A. phagocytophilum* PCR assay results persist up to 42 days despite the high antibody response suggesting that the humoral response is not sufficient to clear the infection (284). It appears that the innate immune mediators used to activate phagocytes to kill other intracellular bacteria (reactive nitrogen intermediates, Toll-like receptor 2 and 4, MyD88, phagocyte NADPH oxidase) do not play a crucial role in *A. phagocytophilum* clearance and may contribute to the observed pathology (301, 303, 311).

## Canine Granulocytic Anaplasmosis

### Clinical Signs

The discrepancy between the high seroprevalence and the relatively low number of sick dogs in endemic areas suggests that most infected dogs remain apparently healthy or develop a mild self-limiting illness (9, 10, 13, 25). The severity of the disease varies from mild subclinical to severe acute forms (24, 34), with severe clinical presentation often associated with co-infections, the immune response of the host and the variability of strains pathogenicity (13, 18, 33, 45).

CGA is a multi-systemic unspecific acute illness characterized by many clinicopathological modifications due to the possible involvement of several body systems. After an incubation period of 1–2 weeks, the most frequently observed clinical signs include fever, lethargy, inappetence or anorexia, weight loss and musculoskeletal pain or discomfort (Table 2) (24, 25, 29, 30, 35, 53, 60, 312, 314). More than 75% of dogs display lethargy and inappetence or anorexia (24, 26, 29, 30, 33, 35). Lethargy has been reported in almost all infected dogs (13, 24–26, 29–31, 33, 35, 45) and was the most frequent clinical signs in several studies (24–27, 29, 33, 35). It was also reported to be disproportionately severe in comparison with the lack of other clinical abnormalities in a case report (247). Fever is both inconstant and variable with frequencies ranging from 46 to 100% **(**24, 33, 35) and values from 39.2 to 41.5°C (25– 27, 30, 35, 246, 251, 252, 257, 258, 261, 314). Fever generally coincides with the peak of bacteremia and lasts less than a week (33). Musculoskeletal pain or discomfort has been described in more than 50% of dogs and manifests in reluctance to move, weakness, stiffness, lameness, and myalgia. However, <10% of dogs have overt joint pain (29, 30). Lameness and joint swelling were reported in 11–34% (25, 34, 35) and 6–62% cases (24, 26, 35) respectively. They are more likely related to neutrophilic inflammation (25, 26, 244, 314, 315), but immune-mediated mechanisms also might be involved (244, 315). In a retrospective study, polyarthropathy (50%) was more frequently observed than monoarthropathy (5%) (27). In a report from California investigating the prevalence of tick-borne infections in dogs with polyarthritis and/or thrombocytopenia, *A. phagocytophilum* was the most frequently detected pathogen (244). Lymphadenopathy, splenomegaly and hepatomegaly were frequent findings in CGA (25, 26, 29, 35, 53, 314, 315). Splenomegaly was reported in 12–100% of naturally infected dogs (25, 26, 35). In canine and murine models of *A. phagocytophilum* infection, lymphadenopathy and splenomegaly are due to reactive lymphoid hyperplasia, with concurrent extra-medullary hematopoiesis in the spleen, enlarged activated lymph nodes and increased numbers of macrophages and plasma cells in the red pulp (284, 285, 314). In experimentally infected dogs, non-specific reactive hepatitis and mild periportal inflammatory lesions were also described (284, 314) and lesions tended to be more pronounced in dogs euthanized in the acute stage (314).

**Table 2:** Clinical signs associated with canine granulocytic anaplasmosis after natural infection and corresponding frequency recorded in several studies.

**Clinical sign**	**Frequency (%)**	**Number of dogs included in the study**	**References**
Fever	84 47 61 46 89 52 100 57 67 60 67	32 49 18 26 18 60 8 28 6 15 61	(26) (31) (25) (33) (27) (45) (24) (13) (312) (60) (35)
Fever, lethargy	88	17	(29)
Fever, lethargy, depression	93	14	(30)
Fever, lethargy, anorexia	51	107	(34)
Lethargy/depression	94 88 74 72 67 81 67 26 50 83 73	18 17 34 18 49 26 60 28 6 63 15	(25) (29) (26) (27) (31) (33) (45) (13) (312) (35) (60)
Inappetence/anorexia	62 33 58 55 29 87 88 50 63 67	34 49 26 60 51 8 17 6 63 15	(26) (31) (33) (45) (13) (24) (29) (312) (35) (60)
Weight loss	25	8	(24)
Pale mucous membrane	28 12 50	18 8 6	(25) (24) (312)
Dehydratation	37	8	(24)
**Musculoskeletal Signs**			
Lameness	32 16 11 50 23 34 16 16 27	34 49 18 26 60 107 28 63 15	(26) (31) (25) (33) (45) (34) (13) (35) (60)
Joint swelling	62 6 19 55 14 33	8 34 26 18 28 6	(24) (26) (33) (27) (13) (312)
Digestive signs	23 13	107 15	(34) (60)
Vomiting	24 11 15 6 50 8	34 18 26 18 6 63	(26) (25) (33) (27) (312) (35)
Diarrhea	9 17 50 14	34 18 6 63	(26) (25) (312) (35)
Abdominal pain	9	34	(26)
Tense abdomen	28 40	18 63	(25) (35)
Lymphadenopathy	32 19 6 13 6	34 26 18 17 63	(26) (33) (27) (29) (35)
Splenomegaly	12 40 13 100 17 84	34 60 17 18 6 57	(26) (45) (29) (25) (312) (35)
Hepathomegaly	8 33	26 6	(33) (312)
Hepatosplenomegaly	7 12	17 57	(29) (35)
**RESPIRATORY SIGNS**			
High respiratory rate	29 2	34 63	(26) (35)
Cough	8 37 3	26 8 63	(33) (24) (35)
Respiratory or urinary tract disease	7	91	(34)
Bleeding disorders	12 13	66 63	(34) (35)
Petechiae	11 3	18 63	(25) (35)
Epistaxis	6 8 6 4 2	18 26 18 28 63	(25) (33) (27) (13) (35)
Melena	6 17	18 6	(25) (312)
Gingival bleeding, hematoma, fresh blood in feces, pulmonary and vaginal hemorrage	2	63	(35)
Neurological signs	7	28	(13)
Left cerebral dysfunction	6	18	(27)
Cervical pain	6 2	18 63	(27) (35)
Proprioceptifon deficit	7	17	(29)
Seizures	15 7 2	40 17 63	(34) (29) (35)
Ataxia	67 7	6 15	(312) (60)
Skin disease	10 12	61 33	(34) (313)

Other clinical signs include gastro-intestinal signs, polyuria, polydipsia, respiratory signs, pale mucous membranes, bleeding disorders, uveitis, scleral congestion, polymyositis, and neurological signs (Table 2) (13, 24, 26, 29, 30, 32, 33, 35, 53, 60, 256, 257, 312, 314, 316). Gastrointestinal signs include diarrhea, nausea, vomiting, and abdominal pain (25–27, 33–35, 312), but their origin is still unknown. In two cases of CGA displaying gastrointestinal signs, associated pancreatitis was suspected based on biochemistry and abdominal ultrasound abnormalities (246, 252). Respiratory signs include dyspnea, tachypnea, and coughing, which is usually infrequent, soft and non-productive (35, 238, 314). One patient displayed coughing and presented interstitial patterns on thoracic radiographs associated with focal alveolar patterns, and showed morulae within neutrophils upon microscopic examination of tracheal lavage specimen (238). Bleeding disorders including petechiae, gingival bleeding, melena, fresh blood in feces, epistaxis, pulmonary hemorrhage, vaginal hemorrhage or hematoma (35) are infrequent in dogs infected with *A. phagocytophilum*, unlike other rickettsial infections, such as *E. canis, A. platys*, and *Rickettsia rickettsii* infections or other infectious diseases, such as aspergillosis, bartonellosis, and leishmaniasis. Indeed, only 3–11% of CGA cases displayed epistaxis (25, 316). In two separate reports, dogs with CGA that presented with epistaxis had mild to moderate thrombocytopenia that could not explain the bleeding disorder. In addition, these dogs were seronegative to *B. burgdorferi, E. canis*, and *Dirofilaria immitis*, but other concurrent diseases were not ruled out. Therefore, other factors than thrombocytopenia may cause epistaxis, such as an infection-induced vasculitis (27, 33). Similarly, another report described two dogs with bleeding disorders associated with *A. phagocytophilum* infection that displayed platelet counts within the reference range (35). Although neurological signs were reported to occur in CGA (35), no studies investigating this association have confirmed the infection by PCR. Moreover, two studies failed to demonstrate an association between *A. phagocytophilum* infection and neurological signs (317, 318). Consequently, *A. phagocytophilum* seems to be a rare cause of neurological disease in dogs and other potential etiologies or concurrent diseases should be ruled out before a final diagnosis of CGA. *Anaplasma phagocytophilum* infection is also suspected to induce skin lesions in dogs (34, 313). In one study that investigated skin-associated lesions in seropositive dogs, four of 12 showed positive DNA amplification from skin lesions. The most frequent lesions identified in these dogs included erythema, papules and plaques that resolved after doxycycline therapy (239**)**. Cutaneous lesions were also present in seropositive but PCR-negative dogs (313). In a previous case report, one dog positive to *A. phagocytophilum* by serologic tests, PCR from blood and post-mortem spleen samples, was presented first for skin problem including pruritus, hair loss and seborrhea in association with regenerative anemia, leukocytosis and thrombocytopenia. *Ehrlichia canis* and *E. chaffeensis* exposure were serologically excluded (256). The lack of typical clinical signs and thrombocytopenia in dogs with PCR-positive skin lesions could be suggestive of a persistent infection as reported in studies in sheep, suggesting that skin could be a site of persistence of *A. phagocytophilum* (313).

### Evolution of the Disease

CGA is currently considered to be an acute disease. Clinical signs usually develop during the bacteremic phase (24, 25, 29, 30) and the duration of the disease is variable. In a retrospective study, the duration of illness ranged from 1 to 14 days with a median duration of 3 days (27). Two studies demonstrated that the majority of dogs were sick for <7 days prior to diagnosis (26, 35). However, the duration of clinical signs ranged from 1 day to 2 months (26). In another report, the duration of illness ranged from 1 to 8 days, but one dog remained infected for a month before the diagnosis was established (30).

Chronic or persistent *A. phagocytophilum* infection has not been demonstrated in naturally infected dogs and is still controversial (2, 24, 53, 319). In contrast, experimental studies showed a persistent infection in dogs for more than several months to almost a year (18, 284, 320–324). These studies support the findings of another report that demonstrated that dogs could have long-lasting infections with acute flare-up (30) whereas another one failed to demonstrate a chronic infection in experimentally infected dogs (324). The results of the latter study differ from those of three other reports in which repeated amplification of *A. phagocytophilum* DNA occurred in some dogs probably because of the differences in the way of inoculation. Indeed, in contrast to the other experimental studies in which the bacterium had been inoculated intravenously to the dogs (18, 284, 320–322), in Contreras et al. (324), dogs were infected through tick bites after *Ixodes* spp. infestation. A 1 year persistence of *A. phagocytophilum* infection has been described in a naturally infected Rhodesian ridgeback dog (53). In addition, some authors consider the possibility of a chronic phase characterized by more localized clinicopathological signs (such as lameness and proteinuria) that could be associated with immune-mediated mechanisms secondary to persistent antigen stimulation (34). Studies on *E. canis* infection in dogs showed that the spleen is probably the organ that harbors bacteria for the longest period and is the best source for the diagnosis of carrier state by PCR (325). Similarly, the spleen remained PCR-positive in monkeys and mice experimentally infected with human strains of *A. phagocytophilum* (299, 326).

The prognosis of the disease in dogs is usually favorable with a rapid remission after doxycycline therapy (24, 26–28, 35, 324). However, some fatal cases have been reported (33, 35, 256, 257). Among the 12 fatality cases reported, five died of immune-mediated hemolytic anemia (IMHA) complicated by disseminated intravascular coagulation (DIC) (33, 256, 257). Two of these dogs were seropositive for *Neorickettsia risticii, R. rickettsii*, and *B. burgdorferi* (33). One was euthanized after 14 days because of IMHA and another one died because of epileptic seizures after 3 days (35).

### Coinfections

Coinfection with multiple VBPs in dogs appears more frequent in endemic areas (9, 13). In a large retrospective serologic study carried out in North America and the Caribbean, exposure to up to five vector-borne pathogens (VBPs) was detected in the same dogs (3). In a kennel of North Carolina, 40% of dogs had serologic evidence of exposure at the same time with *Anaplasma* spp., *Babesia canis, Babesia vinsonii, E. canis*, or *R. rickettsii* (236). In another study, 16.5% of USA dog samples were found to be seropositive for more than one pathogen (119). Two serologic surveys showed that 1.32 and 14.3% of dogs had antibodies against two pathogens in Italy and Morocco, respectively (178, 226). In Tunisia, 22.4% of dogs were seropositive for *E. canis* and *A. phagocytophilum* (224). In Algeria, coinfections by *A. phagocytophilum* and 1–3 other pathogens were higher in stray than client-owned dogs (225). Two studies investigated the association between co-infections with several VBPs and the occurrence on clinical canine leishmaniosis (327, 328) and one reported a statistical association between dogs with clinical leishmaniosis stages III and IV and the seroreactivity to *A. phagocytophilum* in Spain (327). Coinfection with *B. burgdorferi* and *A*. *phagocytophilum* is frequently described in dogs, probably because pathogens are transmitted by Ixodid ticks and maintained in sylvatic cycles with the same rodent reservoir (13, 33, 225, 329–331). In the USA, almost 22% of *A. phagocytophilum*-seropositive samples were also seropositive for *B. burgdorferi* (240). The prevalence of seropositive dogs to both pathogens was as high as 45% (12, 33). The ability of co-infected *I. scapularis* ticks to transmit *B. burgdorferi* and *A. phagocytophilum* was lower compared with transmission of either agent by singly infected ticks (331).

Experimental studies in mouse and human case reports of *A. phagocytophilum* and *B. burgdorferi* coinfection have described an enhanced severity and complexity of clinical signs along with an increased likelihood of disease compared with single infections (13, 329, 330, 332). Similarly, dogs seropositive for both agents (43%) were more likely to display clinical signs than those seroreactive to either *A. phagocytophilum* (25%) or *B. burgdorferi* (9%) (13). Experimental studies in rodents have demonstrated that coinfection modulates the host immune response to *A. phagocytophilum* and the production of interleukins (ILs), decreases IFN-γ levels and the number of CD8^+^ T cells which leads to more severe clinical signs, increases pathogen burdens in blood and tissues, and induces more persistent infections (13, 329, 330, 333). Furthermore, the interaction of both pathogens at the blood-endothelial cell interface seems to be a critical point in pathogenesis (332). Two *in vitro* studies on human blood-brain barrier models showed that *A. phagocytophilum*-infected neutrophils enhanced *B. burgdorferi* migration across both systemic and brain microvascular endothelial cells. Several mechanisms are thought to be involved including impaired phagocytic neutrophil function caused by *A. phagocytophilum*, increased production of vasoactive and pro-inflammatory molecules (IL-6, IL-8, IL-10, tumor necrosis factor alpha, and macrophage inflammatory protein 1α) and the release of matrix metalloproteinases (329, 332). These factors lead to enhanced vascular permeability and inflammatory response in tissues and promote *B. burgdorferi* migration, which results in worsened clinical manifestations (329, 330, 332, 333).

## Laboratory Abnormalities

### Hematological Modifications

Hematological modifications associated with CGA include thrombocytopenia, anemia, leukopenia, and lymphopenia, although variable white blood cells count (WBC) modifications have been described (Table 3) (13, 24–27, 29–33, 35, 45). In experimentally infected dogs, hematological changes usually occurred during the acute stage of infection and normalized a few days after morulae disappeared from blood (302, 314). Suggested mechanisms of cytopenia include cytokine myelosuppression, autoantibodies formation, infection of hematopoietic precursors, and blood cell consumption (especially platelets) (25, 304, 334). Bone marrow aspirates of infected dogs were hyper- or normocellular, with normal, increased, or decreased iron storage, a slight increase in immature erythroid cells, and megakaryocyte and myeloid hyperplasia associated with relative shift toward immature myeloid cells, suggesting impaired myelopoiesis (312, 314).

**Table 3:** Hematological abormalities associated with canine granulocytic anaplasmosis after natural infection and corresponding frequency recorded in several studies.

**Hematological abnormalities**	**Frequency (%)**	**Number of dogs included in the study**	**References**
**Thrombocytes count modifications**			
Thrombocytopenia	95 71 69 89 56 94 65 87 86 86 57 17 86	22 – 49 18 25 18 60 8 7 7 28 6 63	(26) (32) (31) (25) (33) (27) (45) (24) (29) (30) (13) (312) (35)
**Erythrocytes modifications**			
Anemia	47 17 57 82 24 42 3 24 70	34 – 49 11 25 60 15 14 63	(26) (32) (31) (25) (33) (45) (29) (30) (35)
Non regenerative anemia	67 37 50	18 8 6	(27) (25) (312)
Regenerative anemia	27	11	(25)
IMHA	12 24	25 17	(33) (35)
**Leukocytes modifications**			
Leukopenia	9 9 18 55 10 62 7 14	31 – 49 18 60 8 14 63	(26) (32) (31) (27) (45) (24) (30) (35)
Leukocytosis	19 21 61 28 7 7 33 27	31 – 49 25 15 14 6 63	(26) (32) (31) (33) (29) (30) (312) (35)
Lymphopenia	65 12 39 33 100 67 44	31 25 18 60 8 15 63	(26) (33) (27) (45) (24) (29) (35)
Eosinopenia	10 50 9	49 18 63	(31) (27) (35)
Neutropenia	37 7	8 15	(24) (29)
Neutrophilia	19 33 51	31 6 63	(26) (312) (35)
Left shift	28 20 33	26 15 6	(33) (29) (312)
Monocytosis	45 6 33 43	49 18 60 63	(31) (27) (45) (35)
**Morulae**			
Morulae detection	36 56		(29) (24)
	4 56 94	49 18 18	(31) (25) (27)
Percentage of neutrophils with morulae	10–24 7–24 9–32 1–5 0–11	– 5 8 6 35	(29) (30) (24) (312) (316)

Thrombocytopenia is the most common disorder associated with CGA. It has been described in 16.7–95% of natural (13, 24–26, 29, 30, 35) and 100% of experimental infections (302, 314). According to some authors, thrombocytopenia reflects an ongoing immunological response in dogs even when associated with low antibody titers against *A. phagocytophilum* (34). A recent study showed a significant association between thrombocytopenia and high concentrations of circulating immune complexes (CIC), low albumin to globulin (A/G) ratios and an acute phase protein concentration. The importance of thrombocytopenia was emphasized as an indicator of acute anaplasmosis, regardless of antibody titer (28). Therefore, thrombocytopenia is considered the most relevant abnormality in the diagnosis of CGA after morulae detection (13, 24–26, 29, 30). The severity of thrombocytopenia varies from mild to severe and the platelet count has been reported to range from 5,000 to 164,000 cells/μl (24, 25, 29, 30, 35, 314). However, in a report, none of the 12 dogs seropositive to *A. phagocytophilum* had platelet counts lower than 105,000 cells/ml and dogs that were also seropositive to *B. burgdorferi* had a lower median platelet count of 51,000 cells/μl (33). In another study, five of the six CGA cases with significant thrombocytopenia had concurrent diseases (lymphoma and systemic lupus erythematosus) or were serologically positive to *B. burgdorferi* or *E. canis* (29). A prospective study aiming to investigate the presence of bacteria belonging to the genera *Anaplasma* and *Ehrlichia* in 159 blood samples from thrombocytopenic dogs, detected only two *A. phagocytophilum*-PCR positive dogs (335). As it has been described for a wide range of *Ehrlichia* species, CGA-associated thrombocytopenia may be related to platelet consumption due to DIC, immunological destruction, spleen sequestration or production of inhibitory factors (336–338). The organism seems to be able to enter megakaryocytes lineage but without impairment of their ability to produce platelets (339). The mechanism inducing thrombocytopenia seems to be more associated with an inflammatory process rather than with the direct action of *A. phagocytophilum* (34). Destruction of platelets has been suggested as a probable mechanism because of the increased number of both mature and immature megakaryocytes in the bone marrow (302). On the other hand, anti-platelet antibodies have been detected in both human and canine cases, with up to 60 and 80% of patients with CGA and HGA displaying anti-platelet antibodies, respectively (25, 35, 257, 336–338). However, thrombocytopenia usually occurs during the early stages of infection, before antibody detection and has also been described in severely immunocompromised mice due to B or T cell suppression, suggesting that mechanisms other than decreased hematopoietic production or immune-mediated destruction are involved. Increased platelet consumption is also suspected to play an important role (340, 341). *In vitro*, increased production of monocyte tissue pro-coagulant activity in peripheral blood mononuclear cells has been observed, supporting the platelet consumption hypothesis (27, 340).

Anemia is an inconstant hematological finding (34) described in 3–82% of dogs with clinical signs compatible with CGA either seropositive (33), PCR-positive (25, 27, 35, 45), displaying *A. phagocytophilum*-like morulae on fresh blood smear examination (24, 26) or being positive to two (29, 30) or three (31) aforementioned diagnostic methods (Table 3). In a retrospective study, no dogs were anemic, even during the bacteremic phase, but the mean values of hematocrit, hemoglobin concentration and red blood cell counts were significantly lower than in the control group (34). In contrast, three different studies described 63, 67, and 70% of anemic dogs (27, 35, 224). CGA-associated anemia is usually mild to moderate non-regenerative normocytic normochromic resembling anemia of inflammation (24, 27, 29, 31, 302, 312, 314). In nine dogs experimentally infected with *A. phagocytophilum* that developed mild normocytic normochromic anemia, decreased serum iron and total iron-binding capacity were recorded during bacteremia, but levels returned to reference ranges 1 week after the disappearance of morulae (302). In a report, most dogs had mild to moderate anemia with hematocrits ranging from 19 to 39%, but two had severe anemia with hematocrit levels <20% and three had signs of regeneration. Five were suspected to have hemolytic anemia based on increased serum levels of bilirubin but all had negative Coombs tests (25). Regenerative anemia has been less frequently reported, and severe IMHA is an unusual disorder associated with CGA (25, 33, 257, 258). One retrospective duty aiming to investigate infectious causes of lethal immune-mediated anemia in Croatian dogs, only two dogs were found positive to *A. phagocytophilum* DNA and one of these two dogs was also co-infected with *B. canis* (342). Six cases of IMHA in dogs with CGA have been reported in the UK, the USA and Denmark (33, 35, 257, 315). Authors from Germany described the possible occurrence of IMHA in a small number of dogs (25). Others from Belgium described IMHA in a dog with a positive titer to *A. phagocytophilum* and without other concomitant diseases (262). A previous case report described a dog with *A. phagocytophilum* infection (confirmed by positive PCR from blood and post-mortem spleen samples) with regenerative anemia, severe bilirubinuria, and positive test for osmotic resistance of red blood cells. This dog was serologically negative for babesiosis, leptospirosis, *E. canis* and *E. chaffeensis* infections (256). More recently, four dogs had a positive Coombs test among 17 ones that underwent this analysis in a case series on CGA (35). Only one case series has evaluated the prevalence of IMHA associated with CGA. In this study, three dogs had IMHA based on spherocytes in blood smears and/or positive Coombs test, without evidence of abdominal or thoracic neoplasia. However, two dogs had positive antibodies for at least one other TBP including *Neorickettsia risticii* (formerly *Ehrlichia risticii), B. burgdorferi*, and *Rickettsia rickettsii*. The authors emphasized that both *R. rickettsii* and *B. burgdorferi* are not commonly associated with IMHA and *N. risticii* is not yet associated with clinical disease in dogs as suggested by experimental studies (33). In addition, anti-erythrocyte antibodies have been detected in three dogs with CGA in the USA (312). Even if CGA has not yet been proven to be a common cause of IMHA, *A. phagocytophilum* should be included in the differential diagnosis, especially in endemic area (33).

The most diagnostically relevant hematological abnormality in CGA is the identification of *A. phagocytophilum* inclusions within neutrophils during blood smear examinations. Morulae appear classically as basophilic inclusions detectable by light microscopy of peripheral blood smears (41, 234). They are usually present transiently during the bacteremic phase (4–14 days after inoculation) and persist for 4–8 days in experimentally infected dogs (302, 314). Morulae can also be identified from cytocentrifuged synovial fluid, bone marrow aspirates, and they were also present in the abdominal fluid of an unusual CGA case and in the tracheal wash from a dog with respiratory signs (40, 238, 247, 302, 312, 315). The proportion of neutrophils containing morulae in blood smears varies from <1 to 34% (24, 29, 30, 312, 314, 316). In an experimentally study, the most severely affected dogs were those with higher percentage of neutrophils containing morulae and the lowest proportion was recorded in non-febrile dogs (314). In endemic areas, 38% of dogs displaying clinical signs compatible with CGA had morulae within neutrophils (13). Three studies reported that 56%, 94% (25, 27), and 88 to 93% (33) of dogs presented morulae while other reports failed to identify these inclusions (246, 257). It is important to mention that *A. phagocytophilum* morulae cannot be distinguished from those of *E. ewingii*, which can lead to misdiagnosis in the regions where both pathogens are present. Therefore, other methods, such as PCR are needed to confirm the diagnosis (2, 24, 302).

Experimentally infected dogs developed moderate leucopenia (314), but WBC count modifications in naturally infected dogs are considered non-specific and variable, and both decreased and increased WBC counts have been reported (24–27, 29–35, 45). Therefore, the use of the WBC count as a marker of the course of the disease is controversial (34). Lymphopenia is the most frequently reported WBC count abnormality in CGA (24–26, 29, 302, 314). Other reported modifications include leukocytosis, leukopenia, lymphocytosis, eosinopenia, monocytosis, monocytopenia and mild to moderate neutropenia or neutrophilia (24–27, 29–31, 35, 45, 53, 302, 312, 314). Left shift of neutrophils and toxic changes have also been reported to occur with *A. phagocytophilum* infection in dogs (26, 29, 33, 252, 257, 312).

### Serum Biochemistry Profile Modification

Serum biochemistry profile modifications documented in CGA include increased liver enzyme activity, hyperbilirubinemia, hypophosphatemia, hyperproteinemia, hyperglobulinemia, and hypoalbuminemia (Table 4) (24–27, 29–31, 33, 35, 45, 314, 316). A moderate increase in alkaline phosphatase (ALP) was reported in 7–100% of CGA cases and mild to moderate hypoalbuminemia was present in 17–66% (25, 26, 29, 33). In a retrospective study, 30% of dogs displayed a slightly increased alanine aminotransferase (ALT) activity but concurrent diseases had not been ruled out (26). In another report, the most frequent findings in dogs with CGA were increased in liver enzymes and hyperbilirubinemia (35). According to some authors, hypoalbuminemia and hyperglobulinemia might be due to a decreased production of albumin in the liver associated with a rise in α- and β-globulin production (304). In a study investigating serum protein profiles of seropositive and PCR-positive dogs, the major modification was a low A/G ratio (84.4%), mostly in groups with antibody titers higher than 1:1,024. Hyperglobulinemia was due to an increase in the acute phase proteins (α2-, β1-, and β-2 globulin). In the same study, 62 and 71.8% of dogs in the group with lower A/G ratios had thrombocytopenia and clinical signs compatible with CGA, respectively, suggesting an acute infectious process. However, other diseases had not been excluded; hence dysproteinemia could possibly be the result of concurrent diseases (28). Others reported hypergammaglobulinemia as a prominent modification associated with CGA but without exclusion of concurrent diseases (316). Decreased serum levels of urea and hypokalemia have been recorded in 27% of dogs (25) and 27–37% of dogs were reported to have hyperbilirubinemia (25, 26, 35). An increase in serum amylase activity was described in 50% of CGA cases (29). Two case reports described dogs diagnosed with CGA with suspected pancreatitis on the basis of increased serum level of amylase and lipase and clinical signs suggesting pancreatitis (abdominal pain in the pancreatic region of one dog and abdominal ultrasound modifications in the pancreatic region of the other dog) (246, 252). In another previous report, two of seven dogs had increased serum lipase concentrations (24).

**Table 4:** Serum biochemistry abormalities associated with canine granulocytic anaplasmosis after natural infection and corresponding frequencies recorded in several studies.

**Serum biochemistry abnormalities**	**Frequency (%)**	**Number of dogs**	**References**
Hyperproteinemia	12 43	49 62	(31) (35)
Hypoproteinemia	10 20 2	49 11 62	(31) (29) (35)
Hypoalbuminemia	44 29 17 28 44 55 50 62	27 49 23 60 9 18 6 61	(26) (31) (33) (45) (29) (25) (312) (35)
Hyperglobulinemia	50 38	6 61	(312) (35)
Serum protein electrophoresis A/G ratio <0.8	21	145	(28)
CIC	80	204	(28)
Hypophosphatemia	62	8	(24)
Increased ALP	52 59 26 43 75 100 7 67	27 49 23 60 8 9 14 61	(26) (31) (33) (45) (24) (29) (30) (35)
Increased ALT	30 35 18	27 49 62	(26) (31) (35)
Increased bilirubin	37 31 34	27 49 61	(26) (31) (35)
Azotemia	27 3	49 62	(31) (35)
Increased aPTT	60 55	10 29	(25) (35)
Increased PT	30 34	10 29	(25) (35)

*A/G ratio, albumin to globulins ratio; ALP, alkaline phosphatase; ALT, alanine aminotransferase; apt, activated partial thromboplastin time; CIC, circulating immune complexes*.

Prolonged prothrombin time (PT) and activated partial thromboplastin time (aPTT), along with increased fibrin-degradation product concentration and fibrinogen concentration have been reported in some CGA cases (Table 4) (25, 35, 252, 257, 314). DIC was suspected or diagnosed in four dogs; two of which had IMHA (25, 33, 257). Elevated aPTT was also described in one dog with SIRS secondary to *A. phagocytophilum* infection (26). In a recent study on portal vein thrombosis, four of 29 dogs had infectious diseases and one had *A. phagocytophilum* infection (343).

### Urinalysis Modifications

Acute renal failure (ARF) is a complication described in some HGA cases (344, 345). In a recent study, 30.6% of human patients with confirmed *A. phagocytophilum* infection by PCR had abnormalities on urinalysis including hemoglobinuria or myoglobinuria (not distinguished by further analysis). Hemoglobinuria/myoglobinuria could be the precursor of ARF described in severe human cases (346). Experimental studies revealed evidence of *A. phagocytophilum* DNA in the kidneys of three persistently infected lambs and lesions of vasculitis and thrombosis in the kidney of a horse (347, 348). Similarly, one study amplified *A. phagocytophilum* DNA in the kidney of one dog after necropsy (342). CGA is suspected to induce immune-mediated glomerulonephritis (IMGN) likely by vasculitis (349). In contrast to blood modification, urinary abnormalities have not been fully assessed in dogs and only a few reports have described abnormalities in urinalysis (Table 5) (25, 26, 28–30, 33, 312). One such study described the presence of mild to moderate proteinuria, glucosuria, bilirubinuria, hematuria, and epithelial cells in urine sediments. In the same report, only three of eight dogs in which urinalysis was performed were also measured for urine protein to creatinine (UPC) ratios, and one displayed a mild increase (0.88) (25). Another report showed a significant difference in proteinuria between *A. phagocytophilum* seropositive and seronegative dogs (34). In a retrospective study, two dogs displayed proteinuria with UPC ratios of 1.5 and 2.2 (26) and 17% of dogs had proteinuria in another report. In this study the only dog with a UPC ratio higher than one had antibodies against both *A. phagocytophilum* and *B. burgdorferi* (33). More recently, 3% of CGA cases included retrospectively displayed signs of azotemia (35), however other concurrent diseases causing azotemia have not been ruled out. In most studies on CGA, proteinuric dogs were identified mainly on the basis of dipstick and only a few of them underwent UPC measurement. Moreover, urinary tract infection (UTI) was not excluded in all dogs. However, another study demonstrated that 38% of dogs had proteinuria without signs of UTI, which could be compatible with kidney injury (29). Proteinuria due to middle and high molecular weight proteins was found exclusively in 30.5% of *A. phagocytophilum*-seropositive dogs. The authors indicated that proteinuria might be the result of chronic antigenic stimulation and suggested that persistent infection can lead to the development of IMGN (34). In one case of CGA, a persistent proteinuria after 28 days of doxycycline therapy was reported. The dog remained asymptomatic during a 305-day follow up; however, mild proteinuria was still present even with a renin-angiotensin-aldosterone system inhibitor (261). More recently, a case report described a dog with IMGN complicated with systemic hypertension and chronic kidney disease without any identified etiology except an active *A. phagocytophilum* infection on the basis of a very high antibody (1:20,480) titer at first consultation and more than a 4-fold decrease in antibody titer several weeks after (262). Finally, the consensus statement of the American College of Veterinary Internal Medicine (ACVIM) for dogs with suspected glomerular disease recommends serologic screening for anaplasmosis of patients with renal proteinuria in addition to other infectious diseases known to induce proteinuria (350).

**Table 5:** Urinary abormalities associated with granulocytic anaplasmosis after natural infection and corresponding frequency recorded in several studies.

**Urinary abnormalities**	**Frequency (%)**	**Number of dogs**	**References**
Hyposthenuria	12	49	(26)
Proteinuria	15 87 27 38 50 58 40	13 8 23 8 6 58 5	(26) (25) (33) (29) (30) (28) (312)
Glucosuria	12	8	(25)
Bilirubinuria	50 25 50	8 8 5	(25) (29) (312)
Hematuria	87.5 40	8 5	(25) (312)
Hemoglobinuria	60	6	(31)
**Urinary sediment**			
Casts	50	8	(25)
Epithelial cells	75	8	(25)
**Protein electrophoresis**			(28)
LMWP	42	36	
MMWP and HMWP	30	36	

*LMWP, low molecular weight proteins (<66 kDa); MNWP, middle molecular weight proteins (66–76 kDa); HMWP, high molecular weight proteins (>76 kDa)*.

## Conclusion and Futures Perspectives

Understanding granulocytic anaplasmosis is important due to its zoonotic aspect, potential severe outcomes in both dogs and humans, and the possibility of using epidemiological data in canine species as a good estimation of risk for human exposure. The aims of this review were to summarize the wide epidemiological data published on *A. phagocytophilum* in canine species and to describe the clinicopathological aspects of CGA that are available in the few case series and reports. In this manuscript, the authors wanted to gather together all data on *A. phagocytophilum* in dogs that can be valuable for researchers and to highlight the fields where important information is still missing and toward which future research should be focused. Indeed, information regarding the prevalence of *A. phagocytophilum* in some parts of the world, the potential role of dogs as competent reservoir hosts, the possibility of tick species other than *Ixodes* spp. acting as vectors of *A. phagocytophilum* and the implication of the genetic variability in the pathogenesis of the disease with some strains being potentially more virulent for humans is still incomplete or lacking. The pathogenesis of CGA is not fully elucidated too. Finally, some publications on CGA discussed the possibility of a chronic evolution and the association of this disease with serious clinicopathological manifestations with a crucial impact on the prognosis and management, such as immune-mediated hemolytic anemia, glomerulonephritis, and neurological signs that are still incomplete and thus need further investigations.

## Author Contributions

SE wrote the manuscript. SD, LD, LE, MK, and HS drafted and revised the manuscript. All authors have made a substantial, intellectual contribution to the work, and read and approved the final manuscript.

## Conflict of Interest

The authors declare that the research was conducted in the absence of any commercial or financial relationships that could be construed as a potential conflict of interest.
